# Study of Binding Kinetics and Specificity of ^99m^Tc-SSS-Complex and ^99m^Tc-HMPAO to Blood Cells

**DOI:** 10.1155/2018/5603902

**Published:** 2018-10-25

**Authors:** S. Auletta, V. Iodice, F. Galli, N. Lepareur, A. Devillers, A. Signore

**Affiliations:** ^1^Nuclear Medicine Unit, Department of Medical-Surgical Sciences and of Translational Medicine, Faculty of Medicine and Psychology, “Sapienza” University of Rome, Italy; ^2^Department of Nuclear Medicine and Molecular Imaging, University Medical Center Groningen, University of Groningen, Netherlands; ^3^Nuclear Medicine Department, Centre Eugene Marquis, Universitè Europèen de Bretagne, Rennes Cedex, France

## Abstract

Nuclear medicine offers several techniques and procedures to image infection, but radiolabelled autologous white blood cells (WBCs) are still the gold standard. These cells are usually labelled with ^111^In or ^99m^Tc bound to a hydrophobic chelating agent that allows these isotopes to pass through the plasma membrane and enter in the cytoplasm. The most common compound in Europe is HMPAO that efficiently chelates ^99m^Tc. However, up to 20–40% of the complex is released from the cells in the first few hours. The aim of this study was to radiolabel a new compound, (S_3_CPh)_2_ (S_2_CPh)-complex (SSS-complex) with ^99m^Tc and compare its binding kinetics and specificity for WBC with HMPAO. The SSS-complex was labelled with ^99m^Tc and analysed by iTLC and RP-HPLC. In vitro quality controls included a stability assay in serum and saline. Results showed a labelling efficiency of 95 ± 1.2% and 98 ± 1.4% for ^99m^Tc-SSS-complex and ^99m^Tc-HMPAO, respectively (*p*=*ns*). ^99m^Tc-SSS-complex was stable in serum and in saline up to 24 h (94 ± 0.1%). Cell labelling experiments showed a higher incorporation of ^99m^Tc-SSS-complex than ^99m^Tc-HMPAO by granulocytes (62.6 ± 17.8% vs 40.5 ± 15%, *p*=0.05), lymphocytes (59.9 ± 22.2% vs 29.4 ± 13.5%; *p*=0.03), and platelets (44.4 ± 24% vs 20.5 ± 10.7%; *p*=*ns*), but the release of radiopharmaceutical from granulocytes at 1 h was lower for HMPAO than for SSS-complex (10.3 ± 1.9% vs 21.3 ± 1.8%; *p*=0.001). In conclusion, ^99m^Tc-SSS-complex, although showing high labelling efficiency, radiochemical purity, and stability, is not a valid alternative to ^99m^Tc-HMPAO, for example, in vivo white blood cells labelling because of high lymphocyte and platelet uptake and rapid washout from granulocytes.

## 1. Introduction

The early and accurate localization of infectious foci and inflammation is a major challenge in contemporary nuclear medicine. In 1970s, a method for imaging of infections/inflammation, based on the ex vivo labelling of autologous leukocytes with Indium-111 (^111^In), was developed by Thakur and colleagues [1–3]. However, ^111^In showed some drawbacks like poor image quality, unfavorable dosimetry, and cell toxicity, in particular on the white blood cell (WBC) subsets [4–6].

Therefore, new methodologies were developed to replace ^111^In with ^99m^Tc for ex vivo cell labelling, and in 1986, ^99m^Tc-HMPAO entered in clinical practice for WBC radiolabelling and imaging of occult sites of infection [7]. ^99m^Tc-HMPAO is less toxic than ^111^In-oxine to WBC, providing a better image quality and isotope availability. However, if not completely reduced intracellularly, it may be released from cells with time, especially in those patients with impaired redox metabolism (hypovitaminosis, stress, metabolic diseases, drugs, etc.).

Other several agents were tested as an alternative to HMPAO to label WBC. In 90s, ethyl cysteinate dimer (ECD) and d,l-cyclobutylpropylene amine oxime (d,l-CBPAO) were labelled with ^99m^Tc, and their labelling efficiency and stability were compared with ^99m^Tc-HMPAO. Both showed higher radiochemical purity than ^99m^Tc-HMPAO, but only ^99m^Tc-d,l-CBPAO provided a comparable binding to WBC. Despite being reported as a valid alternative to ^99m^Tc-HMPAO, it did not find its place in clinical practice [8, 9]. Pasqualini et al. in 2002 patented [^99m^Tc(S_3_CPh)_2_(S_2_CPh)] (SSS-complex) as a new radiopharmaceutical product for selective labelling of WBC, and in 2003, Mevellec et al. published its synthesis using different methods [10, 11]. They demonstrated that the most efficient labelling method was based on the reaction of a lyophilized formulation of ^99m^Tc-gluconate with the sodium salt of phenyldithiocarboxylic acid. However, no systematic studies have ever been published to show the binding kinetics and specificity of this complex.

Therefore, in the present study, we performed the radiolabelling with ^99m^technetium of SSS-complex and tested its radiochemical purity, stability, binding specificity, and kinetics to different blood cell subsets as compared to ^99m^Tc-HMPAO.

## 2. Materials and Methods

### 2.1. Radiolabelling of SSS-Complex

A technetium-99m reducing kit, containing 4 mg of thin chloride dihydrate, 30 mg of sodium gluconate, 40 mg of potassium oxalate, and 30 mg of ascorbic acid, was reconstituted with 10 ml of saline solution, and 1 ml of this solution was added to freshly eluted ^99m^TcO_4_^−^ (370–720 MBq). The mixture was gently stirred for 10 min at room temperature, and then, 8–10 mg of SSS-complex in 1 ml of saline solution was added. After 15 min of incubation at 100°C, labelling efficiency (LE) and colloid percentages were measured.

### 2.2. In Vitro Quality Controls

Quality controls were performed using both instant thin layer chromatography (iTLC) and reversed phase-HPLC (RP-HPLC). For iTLC, silica-gel strips were used as stationary phase (Pall Life Sciences, Port Washington, NY), whereas a 0.5 M ethanol/toluene/chloroform/ammonium acetate 6 : 3 : 3 : 1 solution was used as mobile phase. In these conditions, it was possible to differentiate pertechnetate (*R*_f_ = 0.5) and the intermediate gluconate complex (*R*_f_ = 0). A mixture of petroleum ether and dichloromethane (6 : 4) was used as the mobile phase to perform quality controls of the ^99m^Tc-SSS-complex (*R*_f_ = 0.7). The strips were analyzed by a radioscanner (Bioscan, Inc, Poway, CA) to calculate the LE. The complex was also analyzed by RP-HPLC using a C18 column (5 mm, 5 *μ*m, 250 × 4.6 mm, Phenomenex, Torrance, CA) and the following mobile phase: H_2_O and THF gradient (0–3 min 70% H_2_O; 3–17 min 100% THF; 17–30 min 70% H_2_O) with a flow rate of 1 ml/min.

Stability assays were performed adding 100 *μ*l of ^99m^Tc-SSS-complex to 900 *μ*l of fresh human blood serum or to 900 *μ*l of 0.9% saline solution. The vials were incubated up to 24 h at 37°C. The LE was measured at 1, 3, 6, and 24 h by iTLC [12].

### 2.3. Radiolabelling of White Blood Cells with ^99m^Tc-SSS-Complex

To evaluate SSS-complex specificity for WBC, whole blood from 4 healthy volunteers (40 ml) was collected and mixed with anticoagulant citrate dextrose ACD (8 ml). The blood was stratified in a centrifuge tube containing 20 ml of lympholyte^®^ (Cedarlane). The vial was centrifuged at 500 g for 20 min at room temperature. After centrifugation, platelets, mononuclear cells (MNCs), red blood cells (RBC), and polymorphonuclear cells (PMNCs) were separated as described in the guidelines [13, 14]. Purity of each cell population was determined by FACS analysis. Platelets, MNCs, and PMNCs (16 × 10^6^ cells) were separately collected and incubated with ^99m^Tc-SSS-complex (74 MBq) under gentle stirring for 10 min at 37°C. Free ^99m^Tc-SSS-complex was removed by centrifugation at 600 g for 10 min and washing with PBS. Cell-bound and free radioactivity was determined by counting the pellet and the supernatant, respectively. The labelling yield was calculated as 100^*∗*^MBq pellet/MBq pellet + MBq supernatants. ^99m^Tc-HMPAO-WBC were prepared as described by EANM guidelines and used as control [13].

### 2.4. Stability Assay

Stability of labelled cells was assessed incubating granulocytes, lymphocytes, and platelets in PBS at 37°C. After 1 h and 3 h, an aliquot from each vial was centrifuged to collect pellet and supernatant that were counted for radioactivity with a single-well gamma counter (Atomlab 500, Biodex) in order to evaluate the radiopharmaceutical elution from labelled cells over time.

The trypan blue exclusion test was also performed in order to verify the viability of each cell population at different time points after labelling.

## 3. Results

### 3.1. Labelling of SSS-Complex

**Figure 1:**
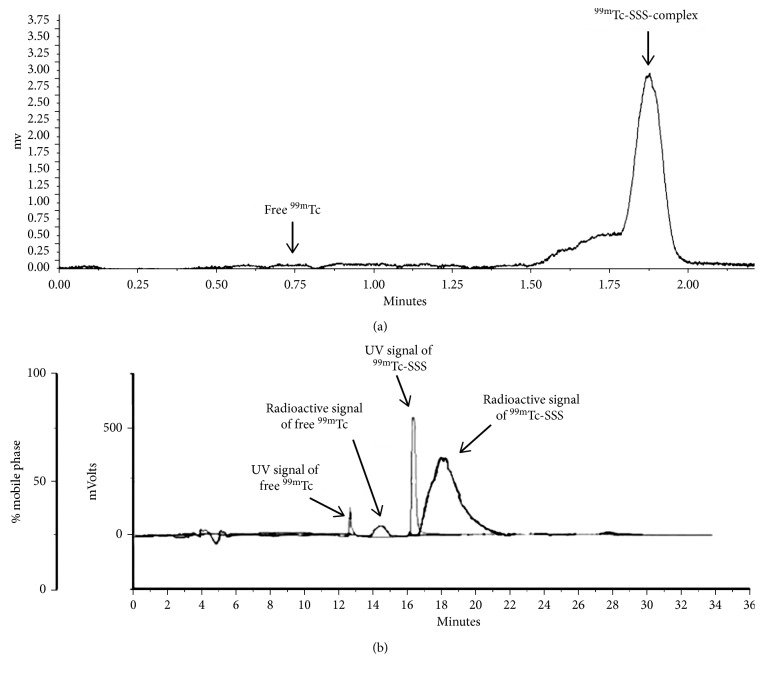
iTLC (a) and HPLC (b) of the radiolabelled compound. Measuring the area under the curve of each peak, the labelling efficiency of ^99m^Tc-SSS-complex is 98.2% as calculated by iTLC and 95.8% as calculated by HPLC. The two peaks (free ^99m^Tc and ^99m^Tc-SSS) at radiochromatogram in b are wider than the UV peaks because the volume of the UV cell is only 10 *µ*l and the volume of radiochromatogram cell is 50 *µ*l for sensitivity reasons.

**Figure 2:**
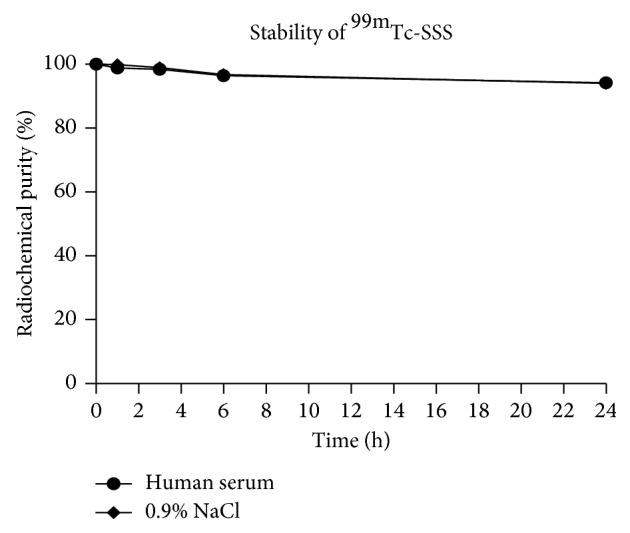
Stability of radiolabelled ^99m^Tc-SSS-complex in saline (diamonds) and in human serum (circles) over time.

The labelling efficiency of ^99m^Tc-SSS-complex was >95%, as assessed by both iTLC and RP-HPLC analysis (Figure 1). In particular, the area below the curve of free ^99m^Tc is 1.8% at iTLC and 4.2% at HPLC. The resulting labelled complex was highly stable in both human serum and saline up to 24 h (94 ± 0.1%) (Figure 2).

### 3.2. Cell Separation and Labelling Technique

**Figure 3:**
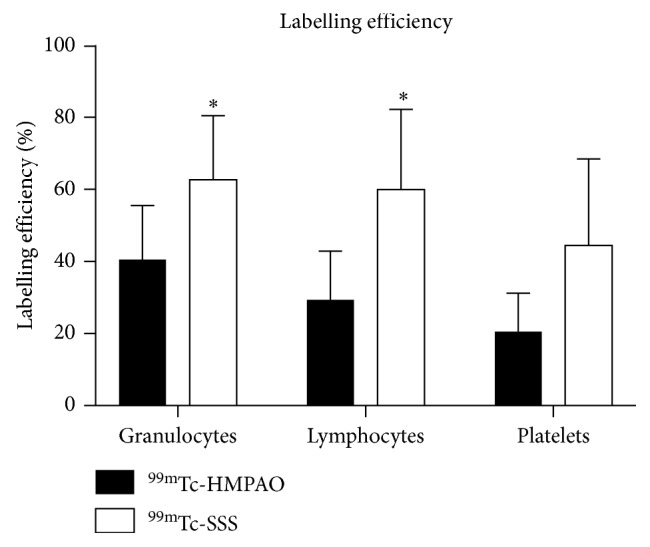
Labelling efficiency of different cell populations. Data are expressed as mean ± SD of four to seven experiments.

FACS analysis revealed a good separation of each blood cell subset, with a purity of >90%. A statistically significant higher accumulation of ^99m^Tc-SSS-complex was observed in each subpopulation as compared to ^99m^Tc-HMPAO after cell labelling (Figure 3). ^99m^Tc-SSS-complex did not show selectivity for any particular blood cell subset as well as ^99m^Tc-HMPAO. In particular, granulocytes were labelled with 62.6 ± 17.8% efficiency with ^99m^Tc-SSS-complex and 40.5 ± 15% efficiency with ^99m^Tc-HMPAO (*p*=0.05); lymphocytes were labelled with 59.9 ± 22.2% efficiency with ^99m^Tc-SSS-complex and 29.4 ± 13.5% efficiency with ^99m^Tc-HMPAO (*p*=0.03); finally, platelets were labelled with 44.4 ± 24% efficiency with ^99m^Tc-SSS-complex and 20.5 ± 10.7% efficiency with ^99m^Tc-HMPAO (*p*=*ns*).

**Figure 4:**
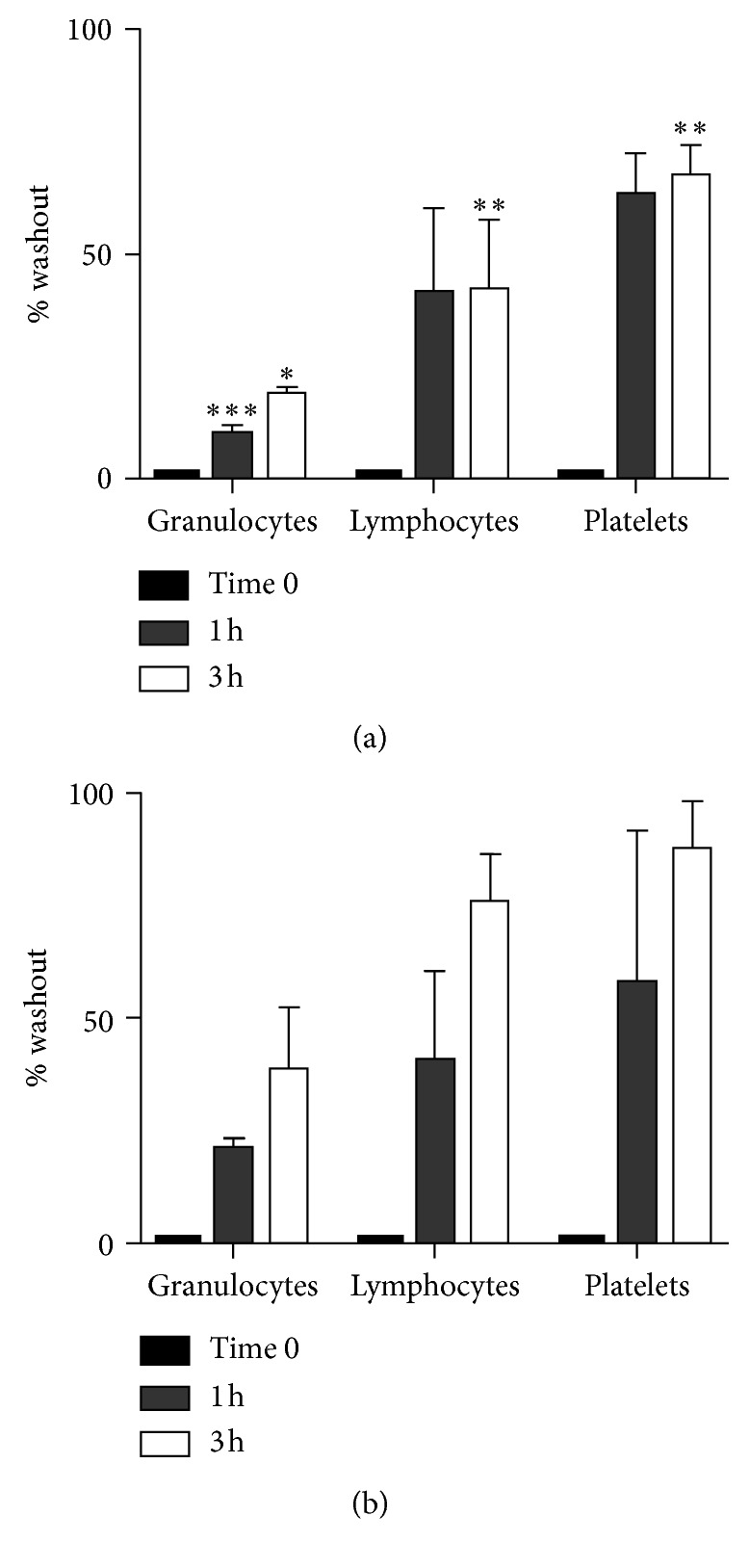
Washout of ^99m^Tc-SSS-complex and ^99m^Tc-HMPAO in different labelled cell populations. All values are normalized to activity at *t* = 0 (black bars) and evaluated at 1 h (grey bars) and 3 h (white bars), expressed as mean ± SD of four to seven experiments.

Regarding ^99m^Tc-SSS-complex, retention experiments showed a rapid decrease of radioactivity in each cell population after 1 h (21.3 ± 1.8%, 40.9 ± 19.6%, and 58.1 ± 33.3%, respectively, for granulocytes, lymphocytes, and platelets), with a further washout up to 3 h (38.6 ± 13.8%, 75.8 ± 10.5%, and 87.6 ± 10.1%, respectively, for granulocytes, lymphocytes, and platelets). ^99m^Tc-HMPAO shows a slower washout from each cells subset at each time point (Figure 4). Particularly, ^99m^Tc-HMPAO showed the following values of washout: 10.3 ± 1.9% (*p*=0.001), 41.6 ± 18.5%, and 63.3 ± 9.1%, respectively, for granulocytes, lymphocytes, and platelets at 1 h, and 18.9 ± 1.6% (*p*=0.05), 42.2 ± 15.4% (*p*=0.005), and 67.4 ± 6.8% (*p*=0.003) at 3 h for granulocytes, lymphocytes, and platelets, respectively.

Labelling of cell subsets with ^99m^Tc-SSS-complex showed no cell toxicity, with more than 99 ± 0.4% viable cells after 24 h.

## 4. Discussion

The development of radiopharmaceuticals to distinguish sterile inflammation from infection is still an open challenge, and it is crucial for the diagnosis of various bone and soft tissue diseases, including osteomyelitis, diabetic foot, immune bowel diseases (IBD), and fever of unknown origin (FUO) too. According to international standardized guidelines, ^99m^Tc-HMPAO-WBC or ^111^In-oxine-WBC are the gold standard to image infection because of their high specificity and rapid clearance from lungs and blood [13, 14]. They specifically accumulate in infectious foci where a neutrophilic infiltrate predominates as a result of migration through the endothelium and basal membrane [15–17]. When using ^111^In-oxine or ^99m^Tc-HMPAO for WBC labelling, a portion of lymphocytes are also radiolabelled. Since lymphocytes are very sensitive to radiation damage [18], it would be ideal to have a Tc-chelating agent that will selectively label only granulocytes in a mixed WBC suspension.

Therefore, the aim of our study was to investigate the properties of a novel compound for granulocyte labelling: the SSS-complex. This was radiolabelled with ^99m^Tc and compared with HMPAO. The labelling procedure of SSS-complex showed *a* >95% LE with negligible amount of ^99m^Tc-colloids and high stability in both human serum and 0.9% NaCl solution.

When compared to ^99m^Tc-HMPAO for WBC labelling, we found a higher labelling efficiency of ^99m^Tc-SSS-complex with respect to ^99m^Tc-HMPAO for granulocyte, lymphocyte, and platelet labelling (Figure 3). But washout from these cells was much faster than ^99m^Tc-HMPAO in all cell populations, reaching 38.6±13.8% of washout from granulocytes at 3 h (Figure 4).

Indeed, granulocytes labelled with ^99m^Tc-HMPAO showed a retention of radioactivity of 90% at 1 h and of 80% at 3 h versus only 80% and 61%, respectively, when labelled with ^99m^Tc-SSS-complex. Washout from lymphocytes and platelets was similar at 1 h between the two radiopharmaceuticals, but higher for ^99m^Tc-SSS-complex at 3 h in both cell subsets.

Based on these results, it appears that ^99m^Tc-SSS-complex cannot substitute ^99m^Tc-HMPAO for selective labelling of granulocytes. It enters into all cell subsets, and most importantly, it is ejected from granulocytes in a higher percentage than ^99m^Tc-HMPAO. This behavior may affect image quality in vivo.

In an attempt to find a better agent for WBC labelling, Capriotti et al. compared ^99m^Tc-HMPAO and ^99m^Tc-stannous colloids in 2004 [19]. In this study, ^99m^Tc-HMPAO showed a lower and significant spontaneous radioactivity release at different time points in all subjects studied, confirming it as the best choice to label WBC.

WBCs were also labelled with ^99m^Tc-liposomes [20] and with ^99m^Tc-P483H [21]. Radiolabelled liposomes showed a minimum release after washings at 2 and 6 h, while ^99m^Tc-P483H showed a radioactivity associated with WBC equal to 76.5%, both obtaining better results than ^99m^Tc-SSS-complex but similar to those achievable with ^99m^Tc-HMPAO.

Since there are no other Tc-chelating agents available for WBC labelling, the only alternative consists in the use of antigranulocyte antibodies [22–24], leaving open doors to the study of new radiopharmaceuticals for bacterial imaging, although radiopharmaceuticals synthetized until now showed several limitations [25, 26].

## 5. Conclusion

^99m^Tc-SSS-complex, although labels white blood cells with high efficiency, showed no selectivity for any particular cell subset, and as the main limiting factor, it showed a high spontaneous release from granulocytes over time. Therefore, in conclusion, ^99m^Tc-SSS-complex cannot be considered as a valid alternative to ^99m^Tc-HMPAO to label granulocytes for in vivo use as an infection seeking agent.

## Data Availability

The data used to support the findings of this study are available from the corresponding author upon request.
